# A Preclinical Study of an ^125^I-Labeled PSMA Ligand for Prostate-Cancer Puncture

**DOI:** 10.3390/ph15101252

**Published:** 2022-10-11

**Authors:** Xiaohui Luan, Haoxi Zhou, Yimin Chen, Xiaojun Zhang, Mengchao Cui, Kuang Chen, Xiaodan Xu, Jinming Zhang, Baixuan Xu

**Affiliations:** 1 Chinese PLA General Hospital Chinese PLA Medical School, Beijing 100853, China; 2 Department of Nuclear Medicine, Chinese PLA General Hospital, Beijing 100853, China; 3 Key Laboratory of Radiopharmaceuticals, Ministry of Education, College of Chemistry, Beijing Normal University, Beijing 100875, China

**Keywords:** ^125^I-PSMA-7, prostate cancer, targeted biopsy, SPECT/CT

## Abstract

Purpose: Prostate cancer (PCa) is characterized by high expression of prostate-specific 1membrane antigen (PSMA), a type II transmembrane protein. Prostate-specific membrane antigen positron emission tomography (PSMA PET) has high sensitivity and specificity and can therefore be potentially used to detect PCa. Exploiting the advantages of PSMA PET imaging, in this study, we aim to develop a novel radiopharmaceutical to facilitate biopsy punching of PCa. Methods: We synthesized a high-affinity radiopharmaceutical of PSMA (^125^I-PSMA-7). We evaluated the properties of ^125^I-PSMA-7, including the purity, stability, affinity, partition coefficient, and toxicity. (PSMA+) 22Rv1 and (PSMA−) PC3 cell lines were used to evaluate ^125^I-PSMA-7 in vitro. BALB/c nude mice bearing 22Rv1 and PC3 xenografts were used for biodistribution and imaging. The uptake of the main organs was evaluated in vivo using single photon emission computed tomography (SPECT). Results: ^125^I-PSMA-7 had a purity of 99.6% and remained stable for seven days and was therefore always safe to use. ^125^I-PSMA-7 had a Ki of 4.037 × 10^−11^ and a partition coefficient of −1.80. The results of in vitro cellular experiments showed a high uptake by 22Rv1 cells (ranging from 2.88 ± 0.14 IA%/10^6^ at 5 min to 61.98 ± 3.43 IA%/10^6^ at 24 h, where the internalization was 46.1% at 1 h and 88.06% at 24 h). However, the uptake of PC3 cells was very low (ranging from 0.34 ± 0.08 IA%/10^6^ at 5 min to 1.60 ± 0.15 IA%/10^6^ at 24 h). The tumors’ uptake of ^125^I-PSMA-7 ranged from 9.02 ± 0.30 ID%/g at 1 h to 4.11 ± 1.04 ID%/g at 7 d and the tumor/muscle ratios and tumor/blood ratios increased over time. In addition, we used γ-counter to measure cpm per milligram of tumor and muscle on days 4 and 7. The background on day 4 is 42 cpm and the tumor is 1739 cpm/mg and the muscle is 45 cpm/mg, and the background on day 7 is 74 cpm and the tumor is 1404cpm/mg and the muscle is 32 cpm/mg. At 1 h post-injection, the high uptake of ^125^I-PSMA-7 resulted in clear delineation of 22Rv1-derived tumors upon imaging. By comparison, 22Rv1-blocking mice took up less ^125^I-PSMA-7. Conclusions: These results show that ^125^I-PSMA-7 is a promising radiotracer that could be used to puncture the prostate. ^125^I-PSMA-7 could be applied to targeted biopsy, reducing the need for saturated biopsy.

## 1. Introduction

PCa is one of the most common malignant tumors and poses a severe threat to men’s health [1]. It has been estimated that in the United States in 2022, nearly 0.27 million new cases of PCa will emerge and nearly 35 thousand men could die from PCa [2]. The development of imaging examinations has resulted in the increasing use of multiparametric MRI (mpMRI) and positron emission tomography (PET) for diagnosing PCa. Whether a repeat biopsy needs to be performed along with an mp-MRI-negative remains controversial [3]. However, the mpMRI currently misses 35% of clinically significant PCa (csPCa) [4]. If a patient’s previous biopsy is negative, but there is constant suspicion of PCa, the European Association of Urology (EUA) guidelines recommend mpMRI as well as a biopsy [5]. Over the past decade, the advent of PSMA-targeted probes has enabled PSMA-targeted PET/CT and PET/MRI to accurately display the location of primary PCa [6,7,8]. PET is superior to mpMRI in terms of diagnostic efficacy, even though PET is not recommended in current clinical guidelines [9].

For the diagnosis or exclusion of PCa, prostate biopsy, with all its limitations, complications and risks, remains a mandatory step. However, up to 30% of csPCa are missed through standard biopsy protocols with 10–12 systematic transrectal ultrasound-guided biopsies (TRUS-GB) [10,11]. The results of using MRI-guided biopsy techniques, such as MRI-guided transrectal-targeted prostate biopsy (MRGB), cognitive target biopsy, and fusion biopsy, were compared against those obtained using TRUS-GB. The results showed that MRI-guided techniques were superior to TRUS-GB, although the difference between the results of the two techniques was not significant [12]. Another study demonstrated that compared with mpMRI, the volume of primary PCa depicted by PSMA PET was highly consistent with postoperative pathologic results7. PSMA PET showed good sensitivity for detecting primary prostatic lesions compared with MRI and TRUS [13,14]. However, a limited number of studies have been performed on the real-time detection of prostate puncture. Therefore, we exploited the advantages of PET precision imaging to evaluate a novel probe synthesized ^125^I-PSMA-7 for the real-time detection of prostate puncture. This ^125^I-PSMA-7 probe was used for in vitro and in vivo studies of PCa puncture.

## 2. Results

### 2.1. Synthesis and Radiochemistry

**Figure 1:**
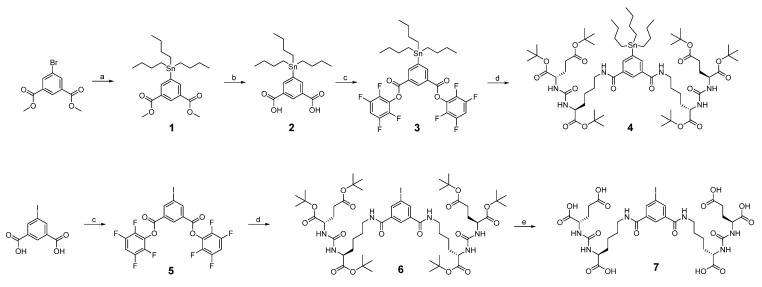
^125^I-PSMA-7 synthesis route map. Reagents and conditions: (**a**) 1, 4-Dioxane, bis (triphenylphoshine)palladium (II) chloride, reflux, overnight; (**b**) LiOH, MeOH/H_2_O, r.t., overnight; (**c**) 2,3,5,6-tetrafluorophenol, DCC (N, N’-dicyclohexylcarbodiimide), r.t., 8 h; (**d**) H-Lys-OtBu-ureido-Glu (OtBu)_2_, CH_2_Cl_2_, Et_3_N, r.t., 8 h; (**e**) CH_2_Cl_2_/CF_3_COOH = 1/1 (*v*/*v*), r.t., overnight.

**Figure 2:**
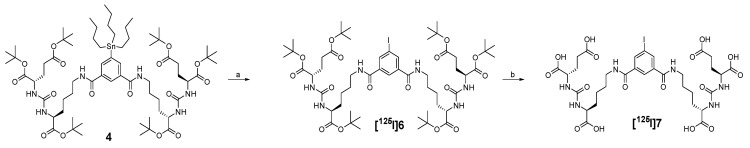
Radiolabeling synthesis route map of ^125^I-PSMA-7. Reagents and conditions: (**a**) [^125^I]NaI, HCl (1 M), H_2_O_2_ (3%), r.t., 20 min; (**b**) CF_3_COOH, 50 °C, 10 min.

The synthetic and radiolabeling steps are shown in Figure 1; Figure 2, respectively.

### 2.2. HPLC Purity Identification and MS Analysis

**Figure 3:**
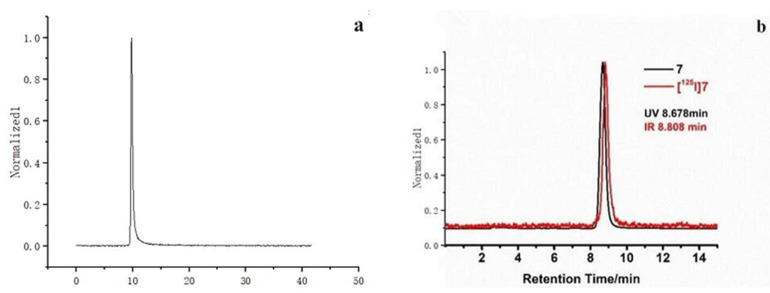
HPLC chromatograms (**a**). The HPLC of ^125^I-PSMA-7. (**b**). The HPLC of a mixture of Compound 7 and ^125^I-PSMA-7.

**Figure 4:**
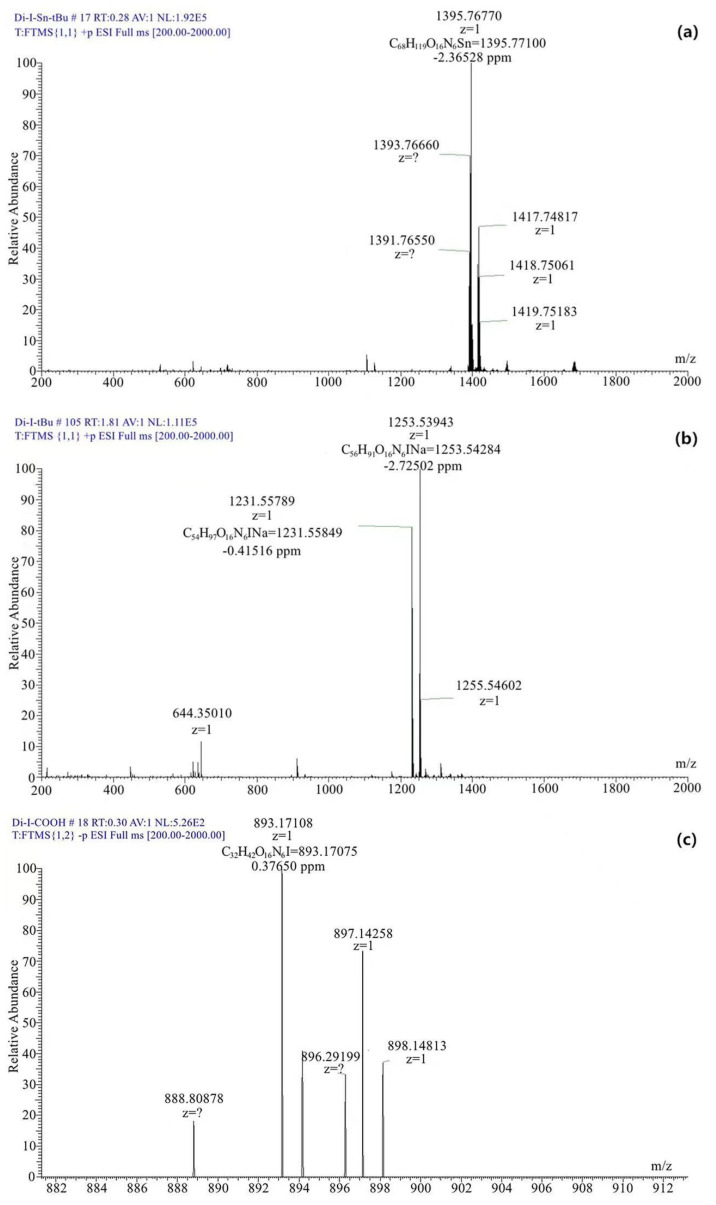
MS analysis (**a**) Compound 4, (**b**) Compound 6, (**c**) Compound 7.

^125^I-PSMA-7 was purified by HPLC to a purity of 99.6% (Figure 3a). The chromatogram of the mixture of Compound 7 and ^125^I-PSMA-7 is shown in Figure 3b. MS analysis was used to determine the molecular weights of three important compounds: Compound 4, Compound 6, and Compound 7. The peaks of these three compounds correspond to 1395 [M+H+] (Figure 4a), 1253 [M+Na+] (Figure 4b), and 893 [M-H-] (Figure 4c).

### 2.3. Determination of PSMA Inhibitory Activity

**Figure 5:**
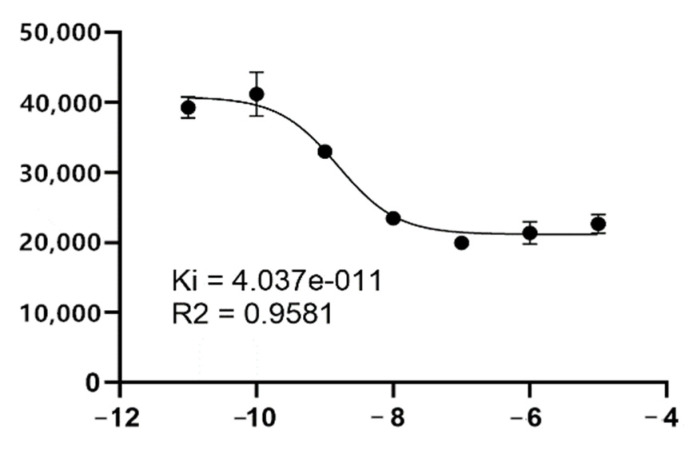
The Ki value of PSMA.

The Ki of ^125^I-PSMA-7 is 4.037 × 10^−11^ (Figure 5) and indicates a high binding affinity to PSMA recombinant protein.

### 2.4. In Vitro Stability and Partition Coefficient

**Figure 6:**
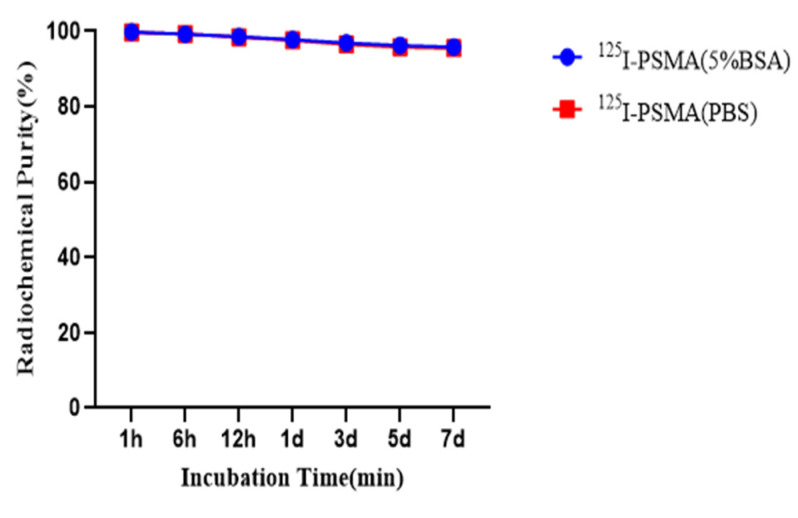
The in vitro stability of ^125^I-PSMA-7 in 5% BSA and PBS solutions.

The log D value of ^125^I-PSMA-7 is −1.80. Performing HPLC on ^125^I-PSMA-7 for 1 h, 6 h, 12 h, 1 d, 3 d, 5 d, and 7 d at room temperature or 37 °C produced chromatograms with a single peak (Figure 6).

### 2.5. Acute Toxicity Test

**Figure 7:**
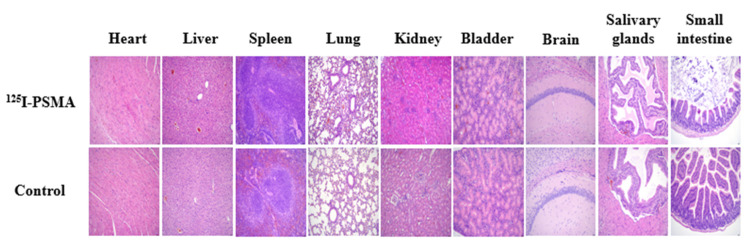
Results of acute toxicity tests on mice. The investigated tissues included the heart, liver, spleen, lung, kidney, bladder, brain, salivary glands, and small intestine. Histopathological analysis of mice by HE staining after a high-dose injection of ^125^I-PSMA-7 (top) and normal saline for the control group (bottom).

Over a 14-day observation period, no significant differences were observed in the diet, activity, mental state, skin or body weight between the experimental and control groups (*p* > 0.05). The results of HE staining (Figure 7) and blood tests were normal (*p* > 0.05).

### 2.6. In Vitro Cellular Experiments

**Figure 8:**
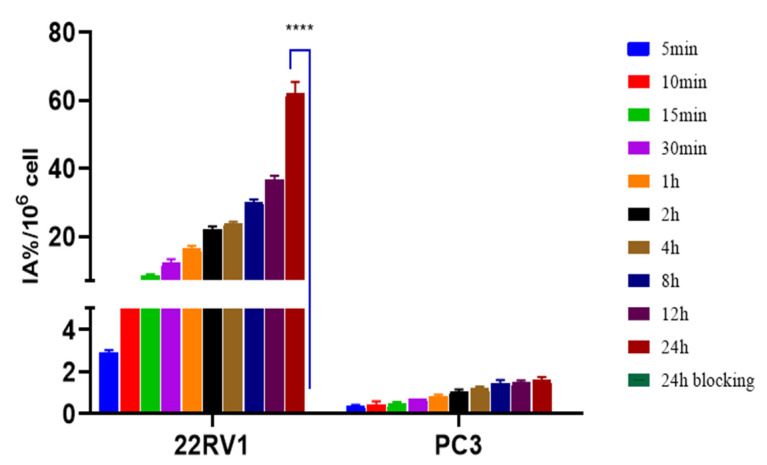
Cell uptake of ^125^I-PSMA-7 in 22Rv1 and PC3 cells at different time points. **** represents a significant difference between the 24h uptake and 24h blockade of the 22RV1.

**Figure 9:**
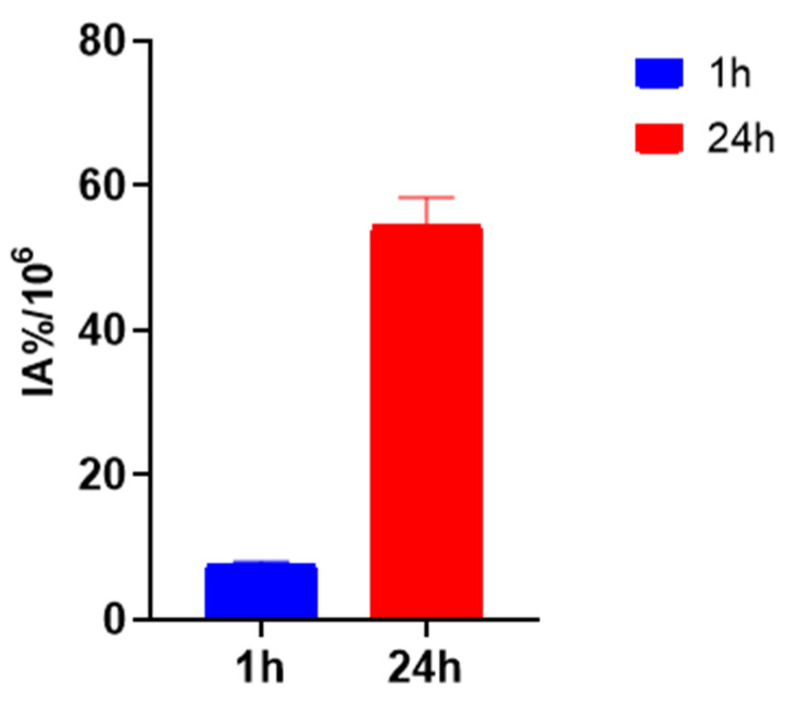
22Rv1 internalization of ^125^I-PSMA-7 at 1 h and 24 h.

The ^125^I-PSMA-7 uptake in 22Rv1 and PC3 is shown in Figure 8. The ^125^I-PSMA-7 uptake of 22Rv1 increased with time from 2.88 ± 0.14 IA%/10^6^ at 5 min to 61.98 ± 3.43 IA%/10^6^ at 24 h. The uptake of 22Rv1 was markedly blocked at 0.10 ± 0.00 IA%/10^6^ by coincubation with ZJ43 for 24 h. The PC3 uptake of ^125^I-PSMA-7 ranged from 0.34 ± 0.08 IA%/10^6^ at 5 min to 1.60 ± 0.15 IA%/10^6^ at 24 h, which was low compared with that of 22Rv1. The rate of 22Rv1 cell internalization was 46.1% at 1 h and 88.06% at 24 h (Figure 9).

### 2.7. Pharmacokinetics

**Figure 10:**
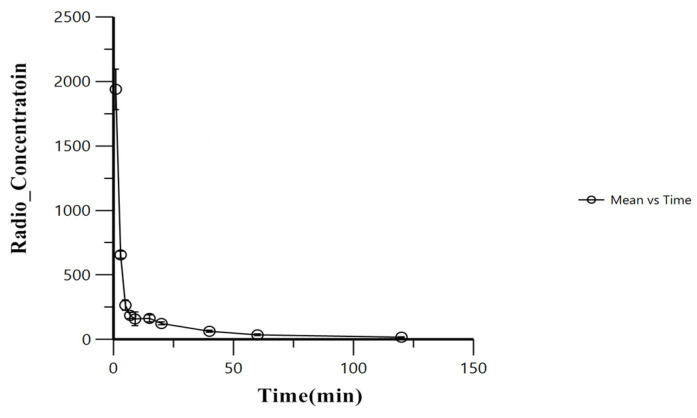
Time–activity curves for blood samples.

WinNonlin was used to plot the time–activity curves for the average blood pharmacokinetics, which are shown in Figure 10. According to the curve type, the distribution and metabolism of the radiopharmaceuticals in the ICR mice was determined to be a two-compartment model. The elimination half-lives of ^125^I-PSMA-7 were 1.04 min (Alpha_HL) and 29.24 min (Beta_HL).

### 2.8. Biodistribution

**Figure 11:**
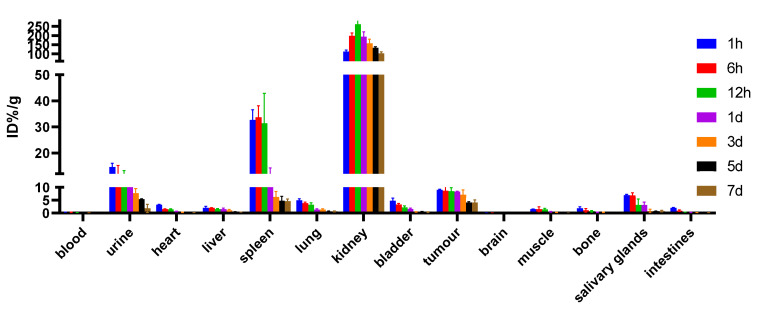
The distribution of ^125^I-PSMA-7 in 22Rv1 tumor-bearing mice after injection of 5.55 × 10^5^ Bq ^125^I-PSMA-7 (values are expressed as the mean ± SD, *n* = 3).

Figure 11 shows that over 7 d, the uptake of ^125^I-PSMA-7 in tumors decreased from 9.02 ± 0.30 ID%/g at 1 h to 4.11 ± 1.04 ID%/g at 7 d, whereas the tumor-to-muscle ratio increased from 5.88 at 1 h to 68.42 at 7 d. The organs with the highest uptake were the kidneys, in which the ^125^I-PSMA-7 level was always high. The uptake in the spleen and salivary glands was also high, whereas low radioactivity accumulation was found for other organs. In addition, Table 1 showed that we used γ-counter to measure cpm per milligram of tumor and muscle on days 4 and 7. The background on day 4 is 42 cpm and the tumor is 1739 cpm/mg and the muscle is 45 cpm/mg, and the background on day 7 is 74 cpm and the tumor is 1404 cpm/mg and the muscle is 32 cpm/mg. The difference between tumor and muscle is striking.

### 2.9. SPECT Imaging

**Figure 12:**
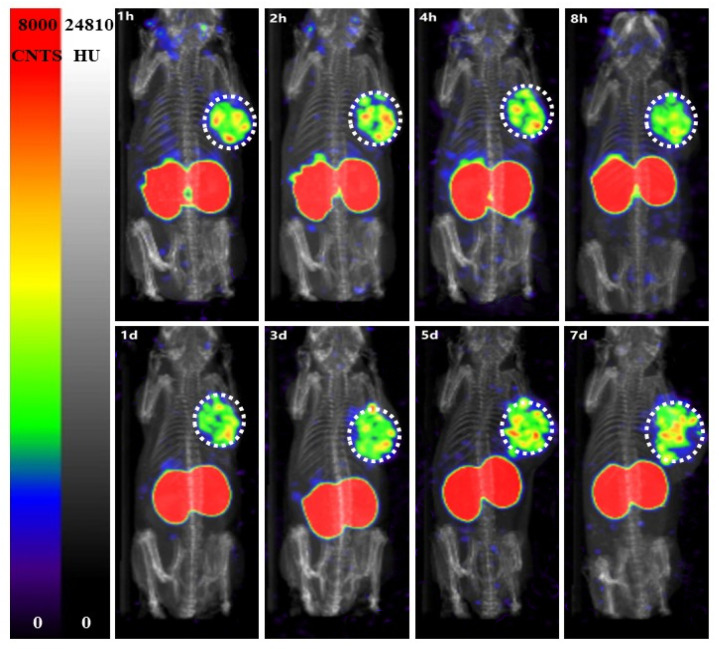
The SPECT images of ^125^I-PSMA-7 on nude mice bearing 22RV1 tumors from 1h to 7 d post-injection. The white dotted circle shows the location of the tumor. As the image shows, the tumor remained visible for seven days.

**Figure 13:**
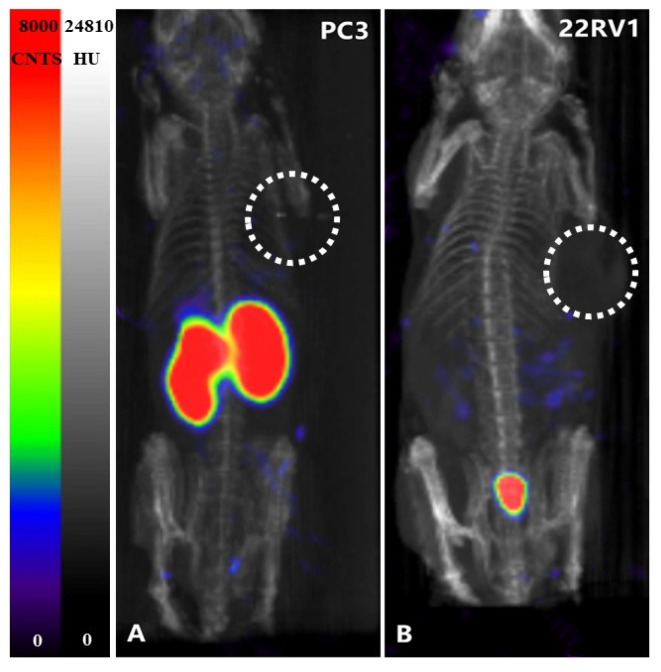
(**A**): SPECT MIP images of PC3 tumor-bearing mice after administration of 1.665 × 10^6^ Bq of ^125^I-PSMA-7 at 60 min; (**B**): Co-injection of 1.665 × 10^6^ Bq i of ^125^I-PSMA-7 and ZJ43 in 22RV1 tumor-bearing mice at 60 min.

Images of ^125^I-PSMA-7 in 22Rv1 and PC3 and 22Rv1-blocking mice are shown in Figure 12 and Figure 13. The 22Rv1 tumor-bearing mice were observed at 1, 2, 4, and 8 h and 1, 3, 5, and 7 d after injection. As the figures show, the tumor and kidneys were always clearly outlined, and the intensity of the background decreased gradually. Low radioactivity was observed for other normal tissues. The images were consistent with the biodistribution data. The tumor and kidneys of the 22Rv1 tumor-bearing mice coinjected with ZJ43 could not be visualized. Although the kidneys of the negative-contrast PC3 tumor-bearing mice could be observed, the tumor was not discernible.

## 3. Discussion

In primary PCa patients, accurate staging and histologic grading are crucial for guiding treatment decisions. PSMA PET outperforms other imaging techniques for localizing the foci of prostate cancer tumors [15,16]. The potential of PSMA-PET for PCa puncture has been evaluated in a few studies. PET and ultrasound images have been fused in some studies, proving that PET-ultrasound fusion targeted biopsy can effectively detect csPCa [17,18]. In other studies, traditional transrectal puncture has been modified: representative procedures are a PSMA target biopsy using a single-puncture percutaneous transgluteal approach and a PSMA-PET-guided robotic-assisted transgluteal prostatic biopsy [19,20]. Robot-assisted radical prostatectomy (RARP) specimens and PCa localization on PET have been found to be highly consistent; thus, a PSMA-guided targeted-prostate biopsy can potentially be an accurate biopsy [19]. However, real-time puncture has been achieved in a few studies. Iodine-labeled radiopharmaceuticals are promising due to a low molecular weight and fast pharmacokinetics [21,22,23,24]. Therefore, we synthesized a novel high-affinity radiopharmaceutical, ^125^I-PSMA-7 to guide real-time puncture.

We used a liquid- phase synthesis method to prepare ^125^I-PSMA-7 with a purity of up to 99.6%. The stability of ^125^I-PSMA-7 in solutions of PBS and 5% BSA was found to be higher than 95% over seven days. The NAALADase method was used to determine the in vitro affinity of ^125^I-PSMA-7. The affinity was on the micromolar scale, indicating that ^125^I-PSMA-7 has a high affinity for PSMA recombinant protein. The results of in vitro cell uptake tests showed a high uptake of ^125^I-PSMA-7 by 22Rv1 cells, where an upward trend was maintained for over 24 h. Blocked 22Rv1 cells took up little ^125^I-PSMA-7. The uptake of ^125^I-PSMA-7 by PC3 cells was also very low. This result demonstrates the targeting specificity of ^125^I-PSMA-7. The internalization rate of 22Rv1 cells was found to be as high as 88% at 24 h, indicating that the agent can be stored in cells for a long time. The short clearance half-life of ^125^I-PSMA-7 of 29.24 min is due to the low molecular weight of the agent. In in vitro distribution experiments on 22Rv1 tumor-bearing mice, tumor uptake remained high up to the seventh day of the experiment. Other organs had a low uptake of ^125^I-PSMA-7, with almost negligible radioactivity on Day 7, whereas the kidney uptake remained high because of the presence of PSMA receptors on the proximal convoluted tubules of the kidney. The results of the tumor-muscle ratio in live animals were also very impressive and increased with time, suggesting that tumors and muscle tissue can be effectively distinguished based on radioactivity. In animal imaging experiments, after injection of ^125^I-PSMA-7 via the tail vein, 22Rv1 tumor-bearing mice showed rapid tumor targeting at 60 min, and tumor uptake was significantly reduced after blockade with ZJ43. By contrast, the tumor uptake of ^125^I-PSMA-7 was very low in PC3 tumor-bearing mice in the control group. The tumor could be clearly imaged over 7 days. Based on these results, ^125^I-PSMA-7 showed a high degree of targeting specificity against prostate cancer in vivo in animals, which was consistent with the results of in vitro experiments. Acute toxicity tests were conducted to assess the safety of the ^125^I-PSMA-7 probe: no tissue or organ damage in mice was observed after a single high-dose injection, demonstrating the low toxicity and safety of ^125^I-PSMA-7. In conclusion, ^125^I-PSMA-7 has a high specificity and can be used for targeted puncture in prostate cancer.

As PSMA-1007 is excreted through the liver and a small fraction of PSMA-1007 is excreted through the urinary system, PSMA-1007 was used in a previous study to guide the puncture. This metabolic pathway rules out the interference of urine in the bladder and can more effectively identify the primary focus of prostate cancer. ^18^F was used to detect radioactivity in tissues instead of ^68^Ga because of a longer half-life [25]. However, ^18^F has a half-life of just under 109.8 min. Due to the limitation of the half-life, the target tissue of a patient can only be punctured approximately 3 h after examination to detect radioactivity, making patient cooperation imperative. Thus, this method for guiding patient puncture is not practical. Therefore, we recommend the use of ^125^I, which has a longer half-life, to ensure that the radionuclide is excreted through the urinary system as late as possible after puncture to avoid contamination; thus, ^125^I with a long half-life can provide good guidance for both first or repeat punctures. In addition, the energy of ^125^I is low, which can ensure the radiation safety of the operator. Additional γ-counter for measuring radioactivity counts is available in the operating room. All these ensure the feasibility of ^125^I-PSMA-7 for prostate-cancer puncture. ^125^I-PSMA-7 cannot be used for SPECT imaging because of its low energy, but we will replace the radionuclide later and label it with ^123^I for clinical application. Iodine-123 has a short half-life and narrow puncture time window after drug injection, while iodine-125 has a long half-life and can be punctured at any time point 34 days after drug injection, so it is more likely to achieve puncture with iodine-125.

## 4. Materials and Methods

### 4.1. General Materials

All chemicals, solvents, and reagents (analytical grade) used for synthesis and analysis were purchased from Maclin Biochemical Technology Co., Ltd. (Shanghai, China). The materials used for cell experiments, such as 24-well plates and the RPMI-1640 medium, were obtained from Gibco Life Technologies (Grand Island, NY, USA). BALB/c male nude mice were purchased from Charles River Laboratories (Beijing, China).

### 4.2. Cell Lines and Mouse Models

The 22Rv1 and PC3 cell lines were obtained from GuYan Biotech Co., Ltd. (Shanghai, China), cultured in the RPMI-1640 medium (Gibco Life Technologies, Grand Island, NY, USA), and supplemented with 1% P/S and 10% fetal bovine serum (FBS). 22Rv1 and PC3 were cultivated in an incubator containing 5% CO_2_ at 37 °C. All cells were grown to 80% to 90% confluence before trypsinization.

All animal experiments conformed to the protocol approved by the Animal Care and Use Committee of the PLA General Hospital. BALB/c male nude mice were purchased from Charles River Laboratories (Beijing, China). The mice were approximately 3–4 weeks old and weighed 13–15 g. Approximately 5 × 10^6^ cells were implanted into the right shoulder of each mouse. Mice were imaged or used in biodistribution assays when the tumor volume reached 200–300 mm^3^. The same implantation method was used for PC3 as for 22Rv1.

### 4.3. Chemical and Radiochemical Syntheses

#### 4.3.1. Synthesis of Compound 1

Dimethyl 5-bromoisophthalate (549 mg, 2.0 mM) was dissolved in 1,4-dioxane by the successive addition of hexabutyldistannane (2325 mg, 4.0 mM, 2 equiv) and bis-triphenylphosphine palladium dichloride (1405 mg, 0.2 mM, 0.1 equiv), followed by reaction overnight at 120 °C under nitrogen protection. The insoluble product was removed by suction and filtration. The crude material was obtained using a rotary evaporator. The crude material was separated and purified by silica column chromatography. Compound 1 (565 mg) was obtained as a colorless liquid in a 58.5% yield. ^1^H NMR (600 MHz, chloroform-d) data: δ 8.58 (t, J = 1.8 Hz, 1H), 8.35–8.26 (m, 2H), 3.95 (s, 6H), 1.57–1.51 (m, 6H), 1.36–1.31 (m, 6H), 1.16–1.09 (m, 6H), 0.89 (t, J = 7.3 Hz, 9H).

#### 4.3.2. Synthesis of Compound 2

Compound 1 (329 mg, 0.68 mM) was dissolved in a methanol solution; lithium hydroxide aqueous solution was added to the resulting mixture, which was reacted overnight at 50 °C. After the reaction was complete, 1 M hydrochloric acid solution was used to neutralize the reaction solution. Methanol was removed using a rotary evaporator. Product 2 (213 mg) was obtained by extraction and filtration as a white solid in a 68.7% yield. ^1^H NMR (400 MHz, DMSO-d_6_) data: δ 13.20 (s, 2H), 8.39 (dd, J = 2.2, 1.1 Hz, 1H), 8.22 (d, J = 1.7 Hz, 2H), 1.51 (q, J = 8.0 Hz, 6H), 1.30 (q, J = 7.3 Hz, 6H), 1.18–1.07 (m, 6H), 0.85 (t, J = 7.3 Hz, 9H).

#### 4.3.3. Synthesis of Compound 3

DCC (408 mg, 1.98 mM) and 2,3,5,6-tetrafluorophenol (284 mg, 1.71 mM) were successively added to a solution of 2 (200 mg, 0.44 mM) in a CH_2_Cl_2_ solution. The resulting mixture was stirred for 8 h at room temperature, and the insoluble product was removed. The solvent was removed by a rotary evaporator to obtain the crude product. The crude material was purified on a silica column to afford 319 mg (96.6%) of 3. Compound 3 was a colorless oily liquid. ^1^H NMR (400 MHz, chloroform-d) data: δ 8.93 (t, J = 1.8 Hz, 1H), 8.66–8.49 (m, 2H), 7.08 (tt, J = 9.8, 7.0 Hz, 2H), 1.61–1.53 (m, 6H), 1.36 (q, J = 7.3 Hz, 6H), 1.24–1.15 (m, 6H), 0.91 (t, J = 7.3 Hz, 9H).

#### 4.3.4. Synthesis of Compound 4

Di-tert-butyl (((S)-6-amino-1-(tert-butoxy)-1-oxohexan-2-yl) carbamoyl)-L-glutamate and triethylamine were added to a solution of 3 (284 mg, 0.38 mmol) in CH_2_Cl_2_ solution. The resulting mixture was stirred for 8 h at room temperature, and the solvent was then evaporated off using a rotary evaporator. The crude product was purified on a silica column to afford 417 mg (80.7%) of 4 as a white solid. ^1^H NMR (400 MHz, DMSO-d_6_) data: δ 8.51 (t, J = 5.6 Hz, 2H), 8.18 (s, 1H), 7.97 (d, J = 1.6 Hz, 2H), 6.28 (t, J = 8.3 Hz, 4H), 4.08–3.91 (m, 4H), 3.31–3.19 (m, 4H), 2.22 (dq, J = 19.3, 10.2, 9.5 Hz, 4H), 1.93–1.78 (m, 2H), 1.66 (dtd, J = 15.0, 9.0, 6.4 Hz, 4H), 1.59–1.49 (m, 12H), 1.41–1.35 (m, 54H), 1.33–1.29 (m, 10H), 1.13–1.07 (m, 6H), 0.85 (t, J = 7.3 Hz, 9H).

#### 4.3.5. Synthesis of Compound 5

Compound 5 was prepared using the same method used to prepare Compound 3 and was obtained as an off-white solid in a 90.3% yield. ^1^H NMR (600 MHz, chloroform-d) data: δ 8.97 (t, J = 1.6 Hz, 1H), 8.83 (d, J = 1.6 Hz, 2H), 7.09 (tt, J = 9.8, 7.0 Hz, 2H).

#### 4.3.6. Synthesis of Compound 6

Compound 6 was prepared using the same method used to prepare Compound 4 and obtained as a white solid in an 88.1% yield. ^1^H NMR (600 MHz, DMSO-d_6_) δ 8.63 (t, J = 5.6 Hz, 2H), 8.30 (t, J = 1.6 Hz, 1H), 8.28 (d, J = 1.5 Hz, 2H), 6.28 (dd, J = 13.4, 8.3 Hz, 4H), 4.06–4.01 (m, 2H), 3.98 (m, 2H), 3.25 (q, J = 6.6 Hz, 4H), 2.28–2.15 (m, 4H), 1.86 (dddd, J = 13.8, 8.8, 6.9, 5.2 Hz, 2H), 1.71–1.60 (m, 4H), 1.53 (tt, J = 14.1, 7.5 Hz, 6H), 1.40–1.36 (m, 59H), 1.32 (m, 4H).

#### 4.3.7. Synthesis of Compound 7

A volume of 3 mL of TFA was added to a solution of 6 (101 mg, 0.082 mM) in CH_2_Cl_2_ (7 mL) under stirring. The mixture was reacted at room temperature overnight, the solvent was evaporated off using a rotary evaporator, and ethyl acetate was then added to precipitate a white solid. This solid was washed three times with ethyl acetate, washed three times with petroleum ether, and dried to obtain 72 mg of Compound 7 as a white solid in a 98.2% yield. ^1^H NMR (400 MHz, DMSO-d_6_) data: δ 12.42 (s, 6H), 8.66 (t, J = 5.6 Hz, 2H), 8.29 (m, 3H), 6.31 (t, J = 8.0 Hz, 4H), 4.15–4.01 (m, 4H), 3.24 (q, J = 6.6 Hz, 4H), 2.31–2.17 (m, 4H), 1.92 (tt, J = 14.1, 6.1 Hz, 2H), 1.69 (tt, J = 14.2, 8.2 Hz, 4H), 1.53 (dh, J = 19.8, 7.0, 6.5 Hz, 6H), 1.33 (p, J = 7.7 Hz, 4H).

### 4.4. Radiochemistry

An [^125^I] NaI solution, 100 μL of HCl (1 M) and 100 μL of a H_2_O_2_ solution (3%) were successively added to a solution of 4 (0.3 mg) in 250 μL of ethanol. The solution was allowed to react for 20 min at room temperature and then neutralized with NaHCO_3_ after which the upper supernatant layer was analyzed by HPLC analysis. HPLC conditions: Venusil MP C^18^ column (Agela Technologies, 4.6 mm × 250 mm), 1 mL/min, UV = 254 nm, CH_3_CN/H_2_O (0.1%TFA) = 90/10. The solvent was removed under a stream of N_2_, and 300 μL of TFA was added to the remaining mixture. The mixture was maintained at 50 °C for 10 min, TFA was removed under a stream of N_2_, and 750 μL of CH3CN/H2O (*v*/*v*, 26/74) were added to the remaining mixture. A liquid-phase analysis and separation were subsequently performed. HPLC conditions: Venusil MP C^18^ column (Agela Technologies, 4.6 mm × 250 mm), 1 mL/min, UV = 254 nm, CH_3_CN/H_2_O (0.1%TFA) = 26/74. The final product, [^125^I]7, was further validated by cosampling analysis with the corresponding stable compound.

### 4.5. Determination of Radiochemical Purity by HPLC and MS Analysis

The purity of ^125^I-PSMA-7 was determined by radio-HPLC. The flow rate was set to 1 mL/min, and the mobile phases were 75% solvent A (H_2_O_2_) and 25% solvent B (acetonitrile + 0.1%TFA). Mass spectrometry was used to analyze and determine the molecular weight of the product.

### 4.6. The Binding Affinity of ^125^I-PSMA-7 and PSMA

The NAALADase inhibition assay was used to determine the Ki of probe [26,27]. A HEPES buffer was used to dilute the PSMA probe to different concentrations: 400 μM, 40 μM, 4 μM, 400 nM, 40 nM, 4 nM, 0.4 nM, and 0.04 nM. Next, 25 μL of PSMA solutions of different concentrations, 25 μL of a NAAG solution (160 μM) and 50 μL of a PSMA recombinant protein solution were successively added to corning black flat bottom 96-well plates.

The mixture was homogenized in a microplate reader, transferred to a shaker incubator, and incubated at 37 °C for 1 h. Then, 100 μL of OPA was added to each well, which was then protected from light for 3 min. The fluorescence intensity of the resulting mixture was measured at Ex/Em = 350/450 nm. The Cheng–Prusoff equation was used to convert the IC_50_ values to Ki values.

### 4.7. In Vitro Stability

An ^125^I-PSMA-7 solution was added to 5% phosphate-buffered saline (PBS) or a bovine serum albumin (BSA) solution. After 1 h, 6 h, 12 h, 1 d, 3 d, 5 d, and 7 d at room temperature or 37 °C, radio-HPLC was used to identify the radiochemical purity of ^125^I-PSMA-7. The experiment was repeated three times.

### 4.8. Partition Coefficient

A volume of 100 μL of an ^125^I-PSMA-7 solution was added to a mixture of 2.9 mL of PBS (0.1 M, pH = 7.4) and 3 mL of n-octanol. The mixture was swirled in a vortex mixer for 2 min at room temperature and then centrifuged at 3000 rpm for 5 min. The resulting mixture was allowed to stand for an adequate period of time to enable separation into layers, a 1000 mL × 3 solution was taken from the top layer, and a 100 μL × 3 solution was taken from the bottom layer. A γ-counter was used to detect the radioactivity of the solutions. The experiment was repeated three times.

### 4.9. Acute Toxicity Test

The PSMA probe was injected through the tail veins of ICR male mice that were 3–4 weeks old and weighed 20–25 g. Based on the specific activity of the iodine compounds of 8.14 × 10^13^ Bq/mM, we calculated the injection dose to be 10 mg/100 kg which was 1000 times compared to the normal dose. The same volume of normal saline was injected into the control group. Following injection, the diet, activity, mental state, skin, and other indicators of the two groups were observed daily. On the 14th day after injection, the mice were sacrificed, and the main organs were removed to observe whether there were significant differences between the two groups in terms of color, shape, texture, and other aspects. The organs were stained with HE.

### 4.10. In Vitro Cellular Experiments

22Rv1 cells and PC3 cells were inoculated in 24-well plates and placed in an incubator containing 5% CO_2_ at 37 °C overnight. The RPMI-1640 medium in the well plates was changed 2 h before performing the cell uptake experiment. First, the solution was removed and rinsed with PBS. Second, ^125^I-PSMA-7 (1.48 × 10^4^ Bq, 100 μL) was added to each well, supplemented with RPMI-1640 medium to 1 mL and placed in an incubator containing 5% CO_2_ at 37 °C. Then, the solution was abandoned at 5, 10, 15, and 30 min and 1, 2, 4, 8, 12, and 24 h, respectively. The cells were washed with 0.5 mL of ice PBS buffer containing 0.2% BSA twice. An NaOH (0.5 M, 0.5 mL) solution was used to lyse the cells in the pore plate, which were then collected in an EP tube. The radioactivity of the cells in each well was measured by a γ-counter and corrected to (%IA)/10^6^. Five parallel wells were set at each time point, and the experiment was repeated three times.

22Rv1 cells were added to an ^125^I-PSMA-7 solution (1.48 × 10^4^ Bq, 100 μL) and incubated for 1 h and 24 h. The cells were washed twice in PBS containing 0.2% BSA; then glycine-HCl (pH = 2.9) buffer solution was added to the cells, which were incubated at 37 °C for 5 min. The cells were washed with ice PBS buffer containing 0.2% BSA, and the radioactivity of the cells was counted using a γ-counter.

A blocking experiment was performed by treating 22Rv1 and PC3 cells with ZJ43 [28,29] for 15 min, followed by the same steps as for the cell uptake experiment.

### 4.11. Ex Vivo Biodistribution and Imaging

Male ICR mice that were 5–6 weeks old and weighed 32–34 g were injected with a ^125^I-PSMA-7 solution (1.48 × 10^6^ Bq, 50 μL) through the tail vein. Blood samples were collected from the tail (5 μL × 3) at 1, 3, 5, 7, 9, 15, 20, 40, 60, and 120 min after injection. The radioactive activity of the blood was measured by a γ-counter. The time–blood radioactivity data of mice were analyzed using a two-compartment model in the Pharmacokinetic software WinNonlin (Delaware, America. 2018-06.). Then, the clearance half-life parameters of the radiopharmaceuticals in the mice were obtained.

To evaluate the distribution of ^125^I-PSMA-7 in tumors and major tissues and organs, biological distribution experiments were carried out in 22Rv1 tumor-bearing mice. Twenty-one 22Rv1 tumor-bearing mice were randomly divided into seven groups (n = 3/group). An ^125^I-PSMA-7 solution (5.5 × 10^5^ Bq, 100μL) was injected into the mice through the tail vein at 1 h, 6 h, 12 h, 1 d, 3 d, 5 d, and 7 d. Three mice were sacrificed at a prescribed time, and the tumors and major tissues and organs (blood, urine, heart, lung, liver, spleen, kidney, bladder, tumor, brain, muscle, bone, salivary-glands, and small intestine) were collected. The tissues and organs were washed and dried with normal saline and weighed using an electronic balance. The radioactive count of the tissues and organs was measured by a γ-counter, and the corresponding radioactive value was calculated after time attenuation correction (%ID/g). In addition, we used γ-counter to measure cpm per milligram of tumor and muscle on days 4 and 7.

22Rv1 tumor-bearing mice and PC3 tumor-bearing mice were used in SPECT (3D whole-body scan, MMP919 collimator) imaging studies. SPECT/CT images were reconstructed by HiSPECT software (California, America. 2016-03.) and analyzed by Vivoquant 2.5 software (Osaka, Japanese. 2015-11.). Three 22Rv1 tumor-bearing mice were randomly selected for injection of ^125^I-PSMA-7 (1.665 × 10^6^ Bq, 100 μL). The mice were placed in an anesthesia box at a prescribed time, and a 3.0% isoflurane/air gas mixture was diffused into the box. The mice exhibited no voluntary activity after 5 min and were placed in a prone position on the scanning bed, during which time anesthesia was maintained using a 1.0% isoflurane/air gas mixture.

In blocking experiments, imaging was performed 60 min after coinjection of ZJ43 (50 mg/kg) with ^125^I-PSMA-7 (1.665 × 10^6^ Bq, 100 μL). SPECT scanning images of the PC3 tumor-bearing mice were used as negative controls.

### 4.12. Statistical Analysis

SPSS 26.0 (New York, America. 2019-05.) and GraphPad Prism 8.0 (San Diego, America. 2018-10.) were used to perform a statistical analysis, where *p* < 0.05 is considered to being statistically significant. Data that conformed to a normal distribution were expressed as the mean ± standard deviation, and other data were expressed using median and quartile spacing. An independent-samples T test was performed on the normally distributed data, and the homogeneity of variance and Wilcoxon rank sum tests were performed on the data inconsistent with a normal distribution.

## 5. Conclusions

In this study, we synthesized a novel radiopharmaceutical ^125^I-labeled PSMA ligand. ^125^I-PSMA-7 has high specificity and sensitivity for PSMA (+) PCa. SPECT/CT images demonstrated that tumors could be clearly detected and ^125^I-PSMA-7 was retained for a long time. Rapid targeting and a high tumor-muscle ratio show that ^125^I-PSMA-7 is a promising radiotracer that can facilitate prostate puncture. Thus, ^125^I-PSMA-7 could be applied to targeted biopsy and reduce the need for saturation biopsy.

## Figures and Tables

**Table 1:** The cpm per milligram of tumor and muscle (cpm/mg).

.	Background	Tumor	Muscle
4d	42	1739	45
7d	74	1404	32

cpm, counts per minutes.

## Data Availability

Data is contained within the article.
